# Predictors of response to Radioactive Iodine Therapy in Intermediate and high risk patients with papillary thyroid carcinoma

**DOI:** 10.1186/s12902-024-01648-8

**Published:** 2024-07-15

**Authors:** Azam Keshavarzi, Fariba Alaei-Shahmiri, Babak Fallahi, Zahra Emami, Mojtaba Malek, Mohammad E. Khamseh

**Affiliations:** 1 Endocrine Research Center, Institute of Endocrinology and Metabolism, Department of Internal Medicine, School of Medicine, Iran University of Medical Sciences, Tehran, Iran; 2 Endocrine Research Center, Institute of Endocrinology and Metabolism, Iran University of Medical Sciences, No. 10, Firoozeh St., Vali-asr Ave., Vali-asr Sq, Tehran, Iran; 3 Research Center for Nuclear Medicine, Tehran University of Medical Sciences, Tehran, Iran; 4 Research Center for Prevention of Cardiovascular Disease, Institute of Endocrinology and Metabolism, Iran University of Medical Sciences, Tehran, Iran

**Keywords:** Papillary thyroid carcinoma, Radioactive Iodine Therapy, Treatment response, predictive factor, Thyroglobulin, locoregional involvement

## Abstract

**Background:**

Radioactive iodine (RAI) therapy is the standard treatment approach after total thyroidectomy in patients with papillary thyroid carcinoma (PTC). We aimed to identify predictive factors of response to the treatment in intermediate and high-risk patients with PTC. In addition, the impact of multiple RAI treatments was explored.

**Methods:**

In a 3-year retrospective study, data from intermediate and high-risk patients with PTC who received RAI therapy following total thyroidectomy, were analyzed by the end of year-one and year-three. Demographic data, tumor size, capsular/vascular invasion, extrathyroidal extension, local or distant metastasis, initial dose and cumulative dose of RAI, serum thyroglobulin(Tg), antithyroglobulin antibody(TgAb), and imaging findings were investigated. Patients with an excellent response to a single dose of RAI treatment, after three years of follow-up were classified as the “Responder group”. Excellent response was defined as stimulated serum Tg less than 1 ng/ml, or unstimulated serum Tg less than 0.2 ng/ml in TgAb-negative patients with negative imaging scans.

**Results:**

333 patient records with a complete data set were analyzed in this study. After three years of initial treatment, 271 patients were non-responders (NR) and 62 were responders (R). At baseline, the median pre-ablation serum Tg level was 5.7 ng/ml in the NR group, and 1.25 ng/ml in the R group (*P* < 0.001). TSH-Stimulated serum Tg greater than 15.7 ng/ml, was associated with response failure even after multiple RAI therapy, AUC: 0.717(0.660–0.774), sensitivity: 52.5%, specificity: 89.47%, *P* < 0.001. On the other hand, multiple RAI therapy was associated with excellent response in 16.2% of the patients. The chance of ER was decreased by 74% if initial post-operation ultrasound imaging confirmed the presence of locoregional involvement, OR 0.26, (95% CI: 0.12–0.55), *P* < 0.001.

**Conclusion:**

Stimulated serum Tg and locoregional involvement after total thyroidectomy are predictive factors of non-response to RAI therapy in intermediate and high-risk patients with PTC. In addition, a minority of patients achieve excellent response after multiple RAI therapy.

## Introduction

Differentiated thyroid cancer (DTC) is the most common endocrine malignancy [1], and its incidence has a rising trend over the past decades all over the world [2]. Papillary thyroid cancer (PTC) represents 85% of DTC [3]. Since 1990, the incidence of thyroid cancer has been the fastest-growing cancer among all types of malignancies [4]. This might be due to over detection of small papillary lesions via diagnostic procedures [1, 5]. In Iran, based on the data from the Iranian National Population-based Cancer Registry (INPCR), it is estimated that thyroid cancers have the highest increase in incidence among all other types of cancers by 2025 [5].

According to the American Thyroid Association (ATA) guideline, further management and follow-up of DTC are based on an estimated risk of recurrence. Hence, the patients are stratified into low, intermediate, and high-risk categories following initial thyroidectomy [6]. Patients in intermediate- and high-risk groups have a higher risk of recurrence and morbidity [6].Total thyroidectomy followed by high-dose I-131 therapy and thyroid hormone suppression is the main treatment strategy widely used for intermediate and high-risk patients with PTC [7]. Most low-risk patients achieve an excellent response to the standard treatment [6]. However; some patients especially in the intermediate- and high-risk groups have evidence of biochemical or structural incomplete response (SIR) [8]. We aimed to identify factors predicting response to RAI therapy by the end of year-three, based on initial response to the RAI treatment according to the ATA response to therapy classification in intermediate- and high-risk patients with PTC. In addition, we explored treatment outcomes in patients with PTC who received multiple RAI therapy within three years after initial thyroidectomy.

## Methods

This retrospective study was carried out between 2007 and 2017. Medical records of intermediate and high-risk patients with PTC were evaluated. Total thyroidectomy was performed for all of the participants. Lymph node dissection was done if needed. Subsequent surgical interventions were performed if locoregional recurrences were detected during follow up. All patients received RAI treatment in a tertiary referral center. The study protocol was approved by the ethics committee of Iran University of Medical Sciences (IR.IUMS.REC.1400.543). The primary endpoint of the study was to evaluate the predicting factors of excellent response to the treatment by the end of year three in patients who had an excellent response to a single dose of RAI treatment by the end of year one. The secondary endpoints were to compare the response to the treatment by the end of year one and year three, as well as to assess treatment outcomes in patients who received multiple RAI treatments by the end of year three.

All patients were treated and followed in a single referral academic center according to the standard protocol based on ATA guideline. Treatment in this study was defined as the use of I-131 to treat known residual locoregional and/or metastatic disease targeting progress-free survival, or even cure [9].

In this study we explored dynamic changes of response-to-treatment categories by the end of year three based on the response categories observed by the end of year one. To overcome the missing data issue, we enrolled patients with the complete available data set. The majority of the participants who fulfilled these criteria had completed data set by the end of year-three.

Patients with intermediate-risk were defined as those with aggressive variants in histology, minor extrathyroidal extension (ETE), vascular invasion, and/or lymph node metastases (more than 5 lymph nodes and/or size between 0.2 and 3 cm). High-risk patients included those with gross ETE, incomplete tumor resection, distant metastases and/or lymph node metastases greater than 3 cm in size [6].

The inclusion criteria were as follows:

Age more than 16 years old, documented diagnosis of PTC based on pathology report on post-thyroidectomy tissue samples, RAI treatment with 100–200 mCi, post-operative ultrasonography, and regular follow-up for at least 3 years following surgery, negative serum Tg Ab.

We excluded patients if they had a history of other primary malignancies, other concurrent treatment modalities such as radiotherapy, chemotherapy, or tyrosine kinase inhibitors in the first three years after diagnosis, incomplete medical records or follow-up during the first 3 years after initial thyroidectomy.

In this study, we used thyroid hormone withdrawal protocol before empirical RAI treatment. Although there are some advantages to dosimetric methods in treatment of patients with PTC and with loco-regional recurrence or metastatic disease [9], the protocol in the referral center was empiric RAI treatment. The dose was chosen according to the ATA risk stratification guideline: 100–150 mCi for intermediate-risk patients and 150 mCi to 200 mCi for high-risk patients in each treatment session [6]. Post-treatment whole-body scan was performed seven days after the RAI treatment. All patients were asked to receive a low iodine diet and follow restricted high-dose iodine exposure (drugs/vitamins, radiographic contrast agent) at least two weeks before RAI treatment or whole body scan. The mean pre-treatment serum TSH level was 37.95 (± 12.05) mIU/L.

### Follow-up

Serum Tg and Tg-Ab were measured on thyroid hormone suppression every 6 months for the first three years following initial thyroidectomy and RAI therapy. In addition, I-131 diagnostic whole-body imaging scan and/or neck ultrasonography was performed if needed. Patients who had biochemical or structural incomplete responses to the treatment were received further RAI treatment. Response to the treatment was evaluated during follow-up visits according to the ATA guideline.

1. Excellent response (ER) was defined as negative imaging scans with either a suppressed (on treatment) serum Tg < 0.2 ng/mL or TSH-stimulated serum Tg < 1 ng/mL, in the absence of anti-TgAb.
2. Biochemical incomplete response was considered as a negative imaging scan with serum Tg ≥ 1 ng/mL on thyroid hormone suppression therapy or stimulated serum Tg ≥ 10 ng/mL, in the absence of TgAb, or rising titer of serum anti-Tg antibody.
3. Structural or functional evidence of disease was defined as stable disease or new locoregional recurrence or distant metastasis with any serum Tg level with or without TgAb. Suspicious features of lymph nodes detected by ultrasound imaging or CT scan include cystic changes, hyperechoic punctuation, and the presence of peripheral vascularization, round-shaped nodes, and the presence of microcalcification [6].
4. Indeterminate response: nonspecific findings on imaging studies, faint uptake in thyroid bed after RAI therapy, non-stimulated serum Tg less than 1 ng/ml, stimulated serum Tg less than 10 ng/mL, stable or decreasing serum anti-Tg antibody in the absence of structural or functional disease, and presence of nonspecific findings on imaging scans that cannot be reliably classified as benign or malignant (include vascular nodules less than one centimeter in the thyroid bed or atypical cervical lymph nodes in ultrasound that have not been biopsied or faint uptake in WBS).

We collected the following information from the medical records: age at diagnosis, gender, type of surgery, tumor size, capsular/vascular invasion/extrathyroidal extension, presence of lymph node or distant metastases, initial TSH-stimulated serum Tg after thyroid hormone withdrawal, Tg Ab, TSH, as well as the dose of RAI, post- RAI treatment whole body scan reports, and the cumulative dose of RAI by the end of year-one and year-three.

### Assessment of the outcomes

Patients were classified according to the treatment response by the end of year-one and year-three. We defined the Responder group (R) as patients with an excellent response (ER) to a single dose of RAI therapy who remained in ER by the end of year-three. The non**-**responder group (NR) comprised patients with incomplete biochemical or structural response, or with indeterminate response to therapy, by the end of year-three.

We also stratified patients for more detailed assessments, into four groups:

#### Group A

patients with ER by the end of year-one and year-three following a single dose of RAI therapy.

#### Group B

patients with ER by the end of year-one, and incomplete biochemical or structural or indeterminate response by the end of year-three.

#### Group C

patients without ER by the end of the year-one, but with ER by the end of year-three.

#### Group D

patients without ER by the end of the year-one, and without ER by the end of year-three despite multiple RAI treatments.

### Statistical analysis

The Statistical Package for the Social Sciences (SPSS) version 24 was used for analysis. Continuous variables were expressed as median (interquartile range, IQR) or median (min-max). Categorical variables were expressed as numbers and percentages. *P*-values ​​less than 0.05 were considered as significant. The groups were compared using t-test, ANOVA or, if necessary, equivalent non-parametric tests. Logistic regression analysis with backward procedure was used to estimate the odds ratio (OR) and 95% confidence interval (CI) of the main predictors of excellent response to RAI treatment. Subsequently, we developed the four- and two-predictor models based on the main predictors determined by logistic regression. The discriminatory power of the predictive models was then compared using C statistics determined by receiver operating characteristic (ROC) analyses. ROC curves and calibration statistics were also used to determine serum Tg cut-off.

## Results

**Fig. 1:**
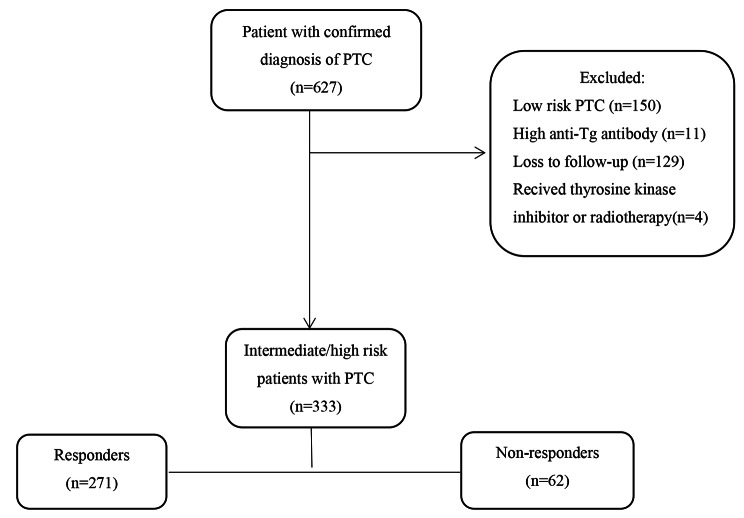
Flowchart of patient selection. Responders: Patients with an excellent response (ER) to a single dose of RAI therapy who remained in ER by the end of year-three. Non**-**Responders: Patients without an excellent response (ER) to a single dose of RAI therapy who remained in ER by the end of year-three

Patient records of 627 individuals were evaluated in this study. After excluding low-risk patients and those with incomplete data, as well as checking for the exclusion criteria, analyses were performed on data from 333 patients (Fig. 1).

The median age of the patients was 39 years. Two hundred and two patients (60.7%) were classified as intermediate and 131(39.3%) as high-risk patients. Baseline characteristics of the study participants are summarized in (Table 1).

**Table 1:** Baseline characteristics of the study participants stratified by treatment response

	Total (*n* = 333)	Non-responders (*n* = 271)	Responders (*n* = 62)	*P*- value
Age (year)	39.0 (30.0–51.0)	38.0 (29.0–50.0)	39.0 (35.5–52.0)	0.284
Female, n (%)	258 (77.5%)	203 (74.9%)	55 (88.7%)	0.019
Lymph node involvement, n (%)^¶^	170 (85.0%)	147 (86.5%)	23 (76.7%)	0.166
Vascular invasion, n (%)	66 (19.8%)	56 (20.7%)	10 (16.1%)	0.419
Capsular invasion, n (%)	88 (26.4%)	69 (25.5%)	19 (30.6%)	0.404
Extra thyroid invasion, n (%)	108 (32.4%)	90 (33.2%)	18 (29.0%)	0.526
Multifocality, n (%)	155 (46.5%)	133 (49.1%)	22 (35.5%)	0.053
Largest tumor dimension (mm)	22 (15–35)	22 (15–35)	25 (12–35)	0.731
Thyroglobulin (ng/ml)	4.40 (1.0–24.0)	5.72 (1.07–34.5)	1.25 (0.39–5.40)	< 0.001
Histopathologic Variant				
Classic	228 (68.5%)	185 (68.3%)	43 (69.4%)	0.972
Follicular	79 (23.7%)	65 (24.0%)	14 (22.6%)	
Aggressive	26 (7.8%)	21 (7.7%)	5 (8.1%)	
Risk stratification				
Intermediate risk	202 (60.7%)	149 (55.0%)	53 (85.5%)	< 0.001
High risk	131 (39.3%)	122 (45.0%)	9 (14.5%)	
Whole body scan findings				
Negative	63 (18.9%)	43 (15.9%)	20 (32.3%)	0.004
Locoregional involvement	256 (76.9%)	214 (79.0%)	42 (67.7%)	
Distant metastasis	14 (4.2%)	14 (5.2%)	0 (0.0%)	
Sonography findings				
Negative	176 (55.9%)	129 (50.2%)	47 (81.0%)	< 0.001
Regional involvement	139 (44.1%)	128 (49.8%)	11 (19.0%)	
Dose of radioiodine therapy at the first session*	150 (100–200)	150 (100–200)	150 (100–175)	0.352
Cumulative dose of RAI therapy at year 1* (mCi)	150 (100–400)	150 (100–400)	150 (100–175)	0.02
1 time, n (%)	299 (89.8%)	237 (87.5%)	62 (100%)	0.001
2 times, n (%)	34 (10.2%)	34 (12.5%)	0 (0.0%)	

Responders are defined as patients who received RAI therapy only for one time and had an excellent response to radioiodine treatment at year 1, without any findings for recurrence/metastasis during 3-year follow-up; Continuous variables are expressed as median (IQR) unless otherwise stated. Categorical variables are presented as n (% within the response group). *, Data are presented as median (min-max); After excluding NX (*n* = 133)^; ¶^, analysed after excluding participants without definite data for lymph node involvement

Multifocality, vascular, and capsular invasions were reported in 155(46.5%), 66 (19.8%), and 88 (26.4%) patients, respectively. Extrathyroidal extension was presented in 108 (32.4%) patients. Distant metastasis was detected in 14 (4.2%) patients at the time of the initial RAI therapy. Moreover, 11 more patients were diagnosed with distant metastasis during the first year of follow-up.

Out of 333 patients, 62 patients were classified as the responder (R) and 271 patients as the non-responder (NR). Lymph node involvement, vascular invasion, ETE and multifocality showed no statistically significant difference between the two groups (All *P*-values > 0.05).

Initial TSH-stimulated serum Tg was significantly different between the two groups when analyzed either as a categorical (1.1 ≤ Tg < 10, ≥ 10 ng/ml) or continuous variable (*P*-value < 0.001). The median initial serum Tg was 5.7 ng/ml in the NR group and 1.2 ng/ml in the R group. Furthermore, 43.5% of the patients in the NR group had serum Tg greater than 10 ng/ml. This was 12.9% in the R group (P-value < 0.001).

Considering response to the treatment by the end of year three26.2% (53/202) of patients with intermediate-risk were classified as R, compared to 6.9% (9/131) of high-risk patients (*P* < 0.001).

Locoregional involvement detected by the first post-RAI treatment whole-body scan images was reported in 79% of patients in the NR group and 67.7% of the R group (*p*-value = 0.004). In addition, initial post-operation ultrasound imaging detected locoregional involvement in 50% of the NR group, and 19% of the R group (*P* < 0.001).

By the end of the year-one, the distribution of response to the treatment was as follows: ER in 35.2% (117/333), incomplete biochemical response in 16.2% (54/333), incomplete structural response in 34.8% (116/333), and indeterminate response in 13.8% (46/333). The main predictors of ER are summarized in (Table 2). The odds of ER were decreased by 74% if post-operation initial ultrasound imaging confirmed the presence of locoregional involvement (OR: 0.27, 95% CI: 0.13–0.56, *P* < 0.001). Moreover, the chance of ER was decreased by 82% if initial serum Tg was greater than 10ng/ml compared to less than 1ng/ml (OR: 0.18, 95% CI: 0.07–0.47, *P* < 0.001). In a subsequent separate analysis in patients with intermediate risk, the presence of locoregional involvement in the ultrasound imaging (OR: 0.30, 95% CI: 0.10–0.92, *P* = 0.04), an initial serum Tg greater than 10ng/ml (OR: 0.09, 95% CI: 0.02–0.41, *P* = 0.002), and the presence of vascular invasion (OR: 0.37, 95% CI: 0.14–0.98, *P* < 0.05) remained as the significant determinants of ER to the initial RAI therapy. Due to a relatively low number of responders in the high-risk group, we were limited to explore the predicting factors of ER in these patients, separately.

**Table 2:** Logistic regression analyses evaluating the main predictors of excellent response to radioiodine treatment by the end of year 1, without any findings for recurrence/metastasis after the 3-year follow-up period - with only one-time RAI therapy (responder group) (*n* = 62)

Parameters	B	S.E.	*P*-value	OR (95% CI)		Standardized B	Standardized OR
Type of surgery							
Total thyroidectomy + lymph node dissection	-0.74	0.32	**0.02**	0.47 (0.25–0.90)		-0.36	0.69
Total thyroidectomy	Ref.						
Baseline thyroglobulin							
Tg ≥ 10	-1.69	0.48	**< 0.001**	0.18 (0.07–0.47)		-1.29	0.27
1 ≤ Tg < 10	-0.42	0.35	0.229	0.66 (0.33–1.30)		-0.32	0.73
<1	Ref.						
Sonography findings							
Regional involvement	-1.33	0.38	**< 0.001**	0.27 (0.13–0.56)		-0.66	0.52
Negative	Ref.						
Vascular invasion							
Yes	-1.07	0.47	**0.02**	0.34 (0.14–0.86)		-0.42	0.66
No	Ref.						
Constant	0.15	0.35	0.67	1.16			

Independents: Age; Sex (Female:1, Male:2); Type of surgery (total thyroidectomy:1, total thyroidectomy + lymph node dissection:2); Vascular invasion (No:0, Yes:1); Capsular invasion (No:0, Yes:1); Extra thyroid invasion (No:0, Yes:1); Focality (unifocal:1, multifocal:2); Variant (classic:1, follicular:2, high risk:3); Whole body scan findings (negative:1, loco-regional involvement/distant metastasis:2); Sonography findings (negative:1, regional involvement: 2); Risk stratification (intermediate risk:1, high risk:2**);** Baseline thyroglobulin (Tg < 1: 1, 1 ≤ Tg < 10: 2, Tg ≥ 10: 3) ; First dose of radioiodine therapy (< 150mCi:1, ≥ 150 mCi:2); B: Estimated coefficient; S.E: Standard error of B; OR: estimated odds ratio (exp (B)); Standardized B: Standardized coefficient B; Standardized OR: Standardized odds ratio

Based on the logistic regression analysis on entire data, we developed a model comprising four variables: type of surgery (total thyroidectomy vs. total thyroidectomy plus cervical lymph node dissection), initial post-operation serum Tg ≥ 10 ng/ml vs. <1ng/ml, vascular invasion (present vs. absent), and post-operation locoregional involvement detected by ultrasound imaging (present vs. absent) (Table 3).

**Table 3:** Models for predicting excellent response to RAI

Models	C-Statistics (95% CI)	SE	*P*-value	*P*-value (vs. Four-predictor model)
Four-predictor model	0.77 (0.71–0.83)	0.03	< 0.001	N/A
Thyroglobulin + Sonography Findings (Two-predictor model)	0.74 (0.67–0.80)	0.03	< 0.001	0.48
Type of surgery	0.56 (0.48–0.65)	0.04	0.13	0.0001
Thyroglobulin	0.68 (61-0.75)	0.04	< 0.001	0.06
Sonography Findings	0.65 (0.58–0.73)	0.04	< 0.001	0.02
Vascular invasion	0.54 (0.46–0.62)	0.04	0.31	< 0.0001

By the end of year-three, the probability of excellent response to a single dose of RAI is predicted to be just 0.9% in patients with all of the following: total thyroidectomy plus cervical lymph node dissection, initial post-operation serum Tg ≥ 10 ng/ml, presence of vascular invasion, and presence of post-operation locoregional involvement (Four- predictor model, Table 3). In patients with initial serum Tg ≥ 10 ng/ml and locoregional involvement detected by ultrasound, the probability of ER is predicted to be 3.4% by the end of year three (Two-predictor model, Table 3).

**Fig. 2:**
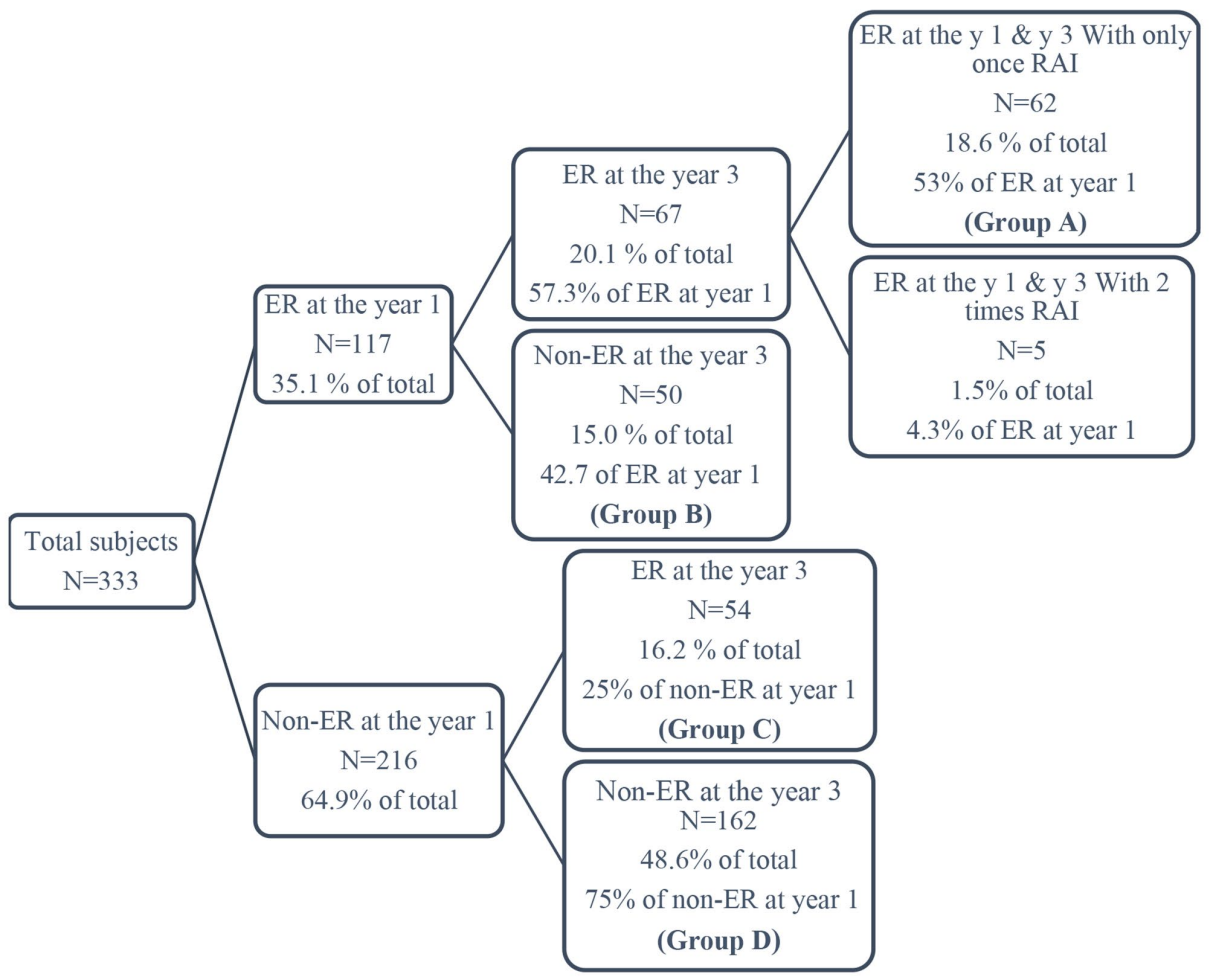
Classification of patients based on response to therapy by the end of the first and third year

**Fig. 3:**
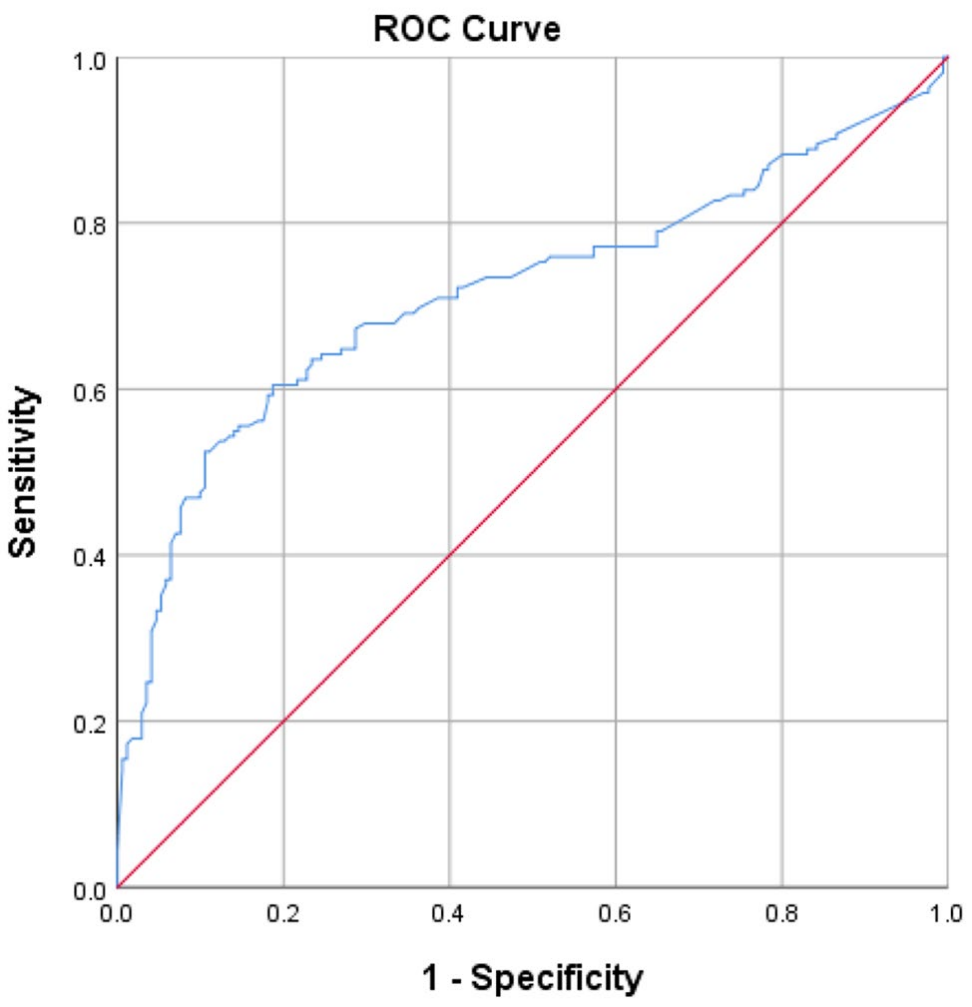
Results of the ROC analysis: the baseline thyroglobulin cut point value of **15.72 ng/ml** [AUC: 0.717(0.660–0.774); sensitivity: 52.5%; specificity: 89.47%, *P* < 0.001] as the best threshold for predicting individuals who did not have an excellent response to RIA therapy neither in year 1 nor after 3 years follow up

We also analyzed the data based on response to the treatment by the end of year-one as well as year- three (Fig. 2). The patients were classified into 4 groups, as mentioned in the method section. Serum Tg at baseline showed a significant increasing trend among the groups (*P* < 0.001). The median serum Tg in groups A, B, C, and D was 1.25 ng/ml, 1.44 ng/ml, 3.85 ng/ml, and 16.25 ng/ml respectively (Table 4). A baseline serum Tg cut-off value of 15.72 ng/ml was found as the best threshold for predicting individuals who did not have an ER either by the end of year-one and year-three (Fig. 3). Furthermore, locoregional involvement detected by post-treatment whole-body scan was found in 67.7% of patients in group A, while it was detected in 81.5% of patients in group D (*P* < 0.001) (Table 4).

**Table 4:** Comparison among characteristics of participants with different responses during a 3-year follow-up period

	Group A (*n* = 62) †	Group B (*n* = 50)	Group C (*n* = 54)	Group D (*n* = 162)	*P*- value	*P*- for trend
Age (year)	39.0 (35.0–52.0)	42.0 (30.0–50.0)	36.0 (28.0–50.0)	38.0 (29.0–51.0)	0.522	0.461
Female, n (%)	55 (88.7%)	45 (90.0%)	38 (70.4%)	115 (71.0%)	**0.003**	**0.001**
Lymph node involvement	23 (76.7%)	22 (81.5%)	31 (83.8%)	92 (88.5%)	0.409	0.092
Vascular invasion, n (%)	10 (16.1%)	12 (24.0%)	11 (20.4%)	31 (19.1%)	0.769	0.875
Capsular invasion, n (%)	19 (30.6%)	15 (30.0%)	9 (16.7%)	42 (25.9%)	0.313	0.401
Extra thyroid invasion, n (%)	18 (29.0%)	10 (20.0%)	22 (40.7%)	58 (35.8%)	0.098	0.114
Multifocality, n (%)	22 (33.3%)	23 (46.0%)	24 (44.4%)	86 (53.1%)	0.119	**0.02**
Largest tumor diameter (mm)	25.0 (12.0–35.0)	22.5 (15.0–35.0)	18.0 (12.0–30.0)	25.0 (15.0–40.0)	0.131	0.181
Thyroglobulin (ng/ml)	1.25 (0.39–5.40)	1.44 (0.60–3.90)	3.85 (1.50–12.7)	16.75(2.0–73.0)	**< 0.001**	**< 0.001**
Thyroglobulin (ng/ml) *	1.25 (0.03-80.0)	1.44 (0.10–33.0)	3.85 (0.18–250.0)	16.75(0.06–3562.0)	**< 0.001**	**< 0.001**
Histopathologic Variant n (%)						
Classic	43 (69.4%)	35 (70.0%)	37 (68.5%)	110 (67.9%)	0.975	0.959
Follicular	14 (22.6%)	10 (20.0%)	14 (25.9%)	40 (24.7%)		
High risk	5 (8.1%)	5 (10.0%)	3 (5.6%)	12 (7.4%)		
Post-RAI therapy whole body scan, n (%)						
Negative	20 (32.3%)	14 (28.0%)	9 (16.7%)	18 (11.1%)	**0.001**	**< 0.001**
Locoregional involvement	42 (67.7%)	36 (72.0%)	43 (79.6%)	132 (81.5%)		
Distant metastasis	0 (0.0%)	0 (0.0%)	2 (3.7%)	12 (7.4%)		
Sonography findings						
Negative	47 (81.0%)	33 (67.3%)	28 (54.9%)	65 (42.8%)	**< 0.001**	**< 0.001**
Regional involvement	11 (19.0%)	16 (32.7%)	23 (45.1%)	87 (57.2%)		
Dose of RAI therapy at the first session*(mCi)	150 (100–175)	150 (100–175)	150 (100–200)	150 (100–200)	0.782	0.346
The number of RAI therapy by the end of year 1						
1 Time, n (%)	62 (100%)	49 (98%)	51 (94%)	133 (82.1%)	**< 0.001**	**< 0.001**
2 Times, n (%)	0 (0.0%)	1 (2%)	3 (5.6%)	29 (17.9%)		
Cumulative dose of RAI during 3 years* (mCi)	150 (100–175)	150 (100–400)	200 (100–650)	325 (100–1025)	**< 0.001**	**< 0.001**
The number and cumulative dose of RAI therapy by the end of year 3						
1 time, n (%) Cumulative dose (mCi)	62 (100%) 150 (125–150)	43 (86.0%) 150 (125–150)	26 (48.1%) 150 (100–150)	39 (24.1%) 150 (125–150)	**< 0.001**	**< 0.001**
2 times, n (%) Cumulative dose (mCi)	0 (0.0%) **-**	6 (12.0%) 312 (300–325)	17 (31.5%) 300 (300–325)	50 (30.9%) 300 (275–325)		
3 or more times, n (%) Cumulative dose (mCi)	0 (0.0%) **-**	1 (2.0%) -	11 (20.4%) 475 (475–550)	73 (45.1%) 550 (450–700)		

**Group A**: ER at year 1 & ER at year 3 with only one-time RAI therapy; †Five subjects with ER at year 1 who had ER at year 3 with two times RIA therapy were excluded from the analysis; **Group B**: ER at year 1 & non-ER at year 3; **Group C**: Non-ER at year 1 & ER at year 3; **Group D**: Non-ER at year 1 & non-ER at year 3; *, Data are presented as median (min-max). *P*-value for difference in thyroglobulin (continuous*)* Group C vs. Group D = **0.003**; *P*-value for difference in thyroglobulin (categorical) Group C vs. Group D **< 0.001**; *P*-value for difference in the number of RAI therapy at year1 Group C vs. Group D = **0.027**; *P*-value for difference *in* cumulative dose of RAI therapy 3 years Group C vs. Group D **< 0.001**; *P*-value for difference in the number of RAI therapy at year *3* Group C vs. Group D **= 0.001**

By the end of year one, 248 patients received 150 mCi or more and 85 patients received less than 150 mCi RAI. There was a significant difference between the cumulative doses received by the end of year one in R group and those in NR group (*P* = 0.02). By the end of year-three, 166 patients received less than 175 mCi RAI and 167 patients received RAI ≥ 175 mCi. The ER was observed in 36.7% of the former and 0.6% of the latter, with *P* < 0.01.

## Discussion

In this retrospective study on intermediate and high-risk patients with PTC, we explored dynamic response to RAI-131 treatment during a three-year follow-up period.

We demonstrated that TSH-stimulated serum Tg, either as a categorical or continuous variable, and locoregional involvement are the two main predictors of treatment response. Stimulated serum Tg level greater than 15.7ng/mL was determined as the best cut-off to predict failure to achieve an ER even with multiple RAI therapy.

Previous studies demonstrated that preablation serum Tg has a high predictive value for the prediction of recurrence and/or treatment failure [3, 10]; however, the optimal cut-off value is different among the studies [7, 11–14]. It is worth mentioning that other factors might affect serum Tg. These include but are not limited to Tg assay techniques. One needs to consider important clinical variables such as patient population, treatment protocol, and follow-up duration.

A study conducted by Prpic et al. on low to intermediate-risk patients with DTC showed preablation Tg/TSH ratio > 0.126 mg/U, Tg level more than 2.4 ng/ml in patients with N1a disease and lymph node capsular invasion have a higher risk of I-131 ablation failure after 6–8 months of initial treatment [15].

Park et al. reported 132 patients with PTC who received 100–200 mCi I131; preablation serum Tg higher than 10 ng/ml was associated with a 25 times greater chance of treatment failure compared with serum Tg less than10 ng/ml in the first follow-up study after mean time interval 7 months. The study was conducted on a wide spectrum of low to high-risk patients without distant metastasis [7].

Matthews et al. showed that Preablation serum Tg equal to or greater than 27.5 µg/l, was associated with a 4.5 times increased relative risk of recurrence at least 12 months after initial RAI treatment in patients with T1-T4, N0-N1 DTC without primary distant metastasis [13]. This cut-off was also reported by Heemstra et al., that stimulated serum Tg greater than 27.5 µg/l is an independent prognostic factor for disease-free remission and mortality [16].

Most of the previous studies have evaluated early treatment response within a short period of 6 to 12 months following RAI treatment. This study aimed to explore dynamic changes in response-to-treatment categories by the end of year three based on the response categories observed by the end of year one. Hence, we identified patients with ER by the end of year -one and then explored factors predicting the ER in those who remained in this response category by the end of year three.

Another study was performed on 452 patients with DTC (TNM staging 1 to 4) who received 30 to 200 mCi RAI and were followed for a median duration of 38 months. Preablative stimulated serum Tg level of 26.75 ng/ml had a high NPV (negative predictive value) of 96.99% for predicting the SIR. Moreover, in a meta-analysis performed by Webb et al. the preablation serum Tg level lower than 10 ng/ml had an NPV of 94% for persistent and recurrent DTC across a broad spectrum of patients with PTC [17]. In our study, we defined ER based on both structural and biochemical definitions as recommended by the international guidelines. Stimulated serum Tg cut-off of 15.72 ng/ml had a PPV of 82.5%, and a NPV of 66.5%.

Our result showed that stimulated serum Tg before treatment higher than 15.72 ng/ml predicts poor therapeutic response even after multiple RAI therapy. This underscores the importance of individualized interventions in patients with PTC. On the other hand, locoregional involvement detected by pre-ablation ultrasound imaging was reported in 50% of NR.

In addition, post-treatment whole-body scan (RxWBS) detected locoregional involvement and distant metastasis in 79% and 5% of the NR group, respectively.

Lateral neck uptake in RxWBS is usually associated with lymph node involvement, although the effectiveness of RAI treatment in such cases is not clear. In a study of 51 patients with PTC and LN metastasis, 65% of patients had detectable disease even after seven years of follow-up [18]. In another study on 95 patients with DTC who had evidence of persistent cervical lymph node metastasis on RxWBS, followed for a mean of 6.8 years, most patients (82%) were free of disease, of course, 47% of them, received 2–4 times of RAI therapy and 15% required surgical re-interventions [19].

We observed that the likelihood of achieving an ER to initial RAI treatment was higher among patients with intermediate-risk compared to those in the high-risk group. Given this finding, which is in line with the previous reports in the literature [6], we examined the predicting factors of ER in the study group by the risk stratification. Despite a limitation in performing a separate analysis in high-risk group, the results of our complementary analyses highlighted the locoregional involvement and an initial serum Tg greater than 10ng/ml as the main determinants of ER in patients with intermediate risk, as well. In line with our findings, one study on intermediate-risk patients with PTC and with ER to initial treatment (total thyroidectomy and RAI therapy with 100–150 mCi), explored the recurrence rate after a median follow-up of 42 months. Lepoutre-Lussey et al. showed that locoregional involvement detected by post-operation ultrasound combined with pre-ablation Tg over 10 ng/ml significantly predicted persistent disease (*P* value < 0.0001) in intermediate and high-risk patients with PTC after a median follow-up-of-41-months. Tumor stage, male gender, and age were also significantly associated with persistent disease. Combination of normal ultrasound and stimulated Tg lower than 10 ng/ml reduced the 5-year recurrence risk to less than 6% [20].

Additionally, findings indicate that age, gender, multifocality, and ETE, were not associated with the response to treatment. Age at diagnosis is not included in the ATA risk stratification system. However, it is considered a main predictor in the American Joint Committee on Cancer Tumor-Node-Metastasis (AJCC-TNM) staging system for mortality. Some previous studies reported a cut-off age of 55years as a strong predictor of incomplete responses [21] or locoregional recurrence [22]; however, Prpic et al. reported patients younger than 53 years have a higher risk of I-131 ablation failure [15]. On the other hand, other studies found no significant association between age and incomplete response in low to high-risk patients with DTC [23, 24]. Moreover, gender was not a predictor of response in our study. In line with our results, previous studies also found no significant association between gender and response to the treatment [10, 23] or recurrent disease [25]. However, an increase in the risk of persistent disease is reported in male patients with PTC [26].

It has been reported that the presence of more than five metastatic cervical lymph nodes following prophylactic lymph node dissection is an independent risk factor for treatment failure [10], or recurrent disease [22, 27]. However, these reports mostly included low-risk patients with stage 1 or those with lateral lymph node involvement [11, 26]. In our study, prophylactic lymph node dissection was not performed for all of the patients. This might explain the lack of association between lymph node involvement and response to treatment. Multifocality is also considered as a risk factor for treatment failure [1], or recurrence [28, 29] of DTC, however, its prognostic significance has not been proven in the other studies [10, 12, and 23]. It should be mentioned that some studies highlighted the size of the lesion, namely greater than one cm, as a prognostic factor [27, 28]. As multifocal lesions greater than one cm were reported in a minority of our patients, this did not reach statistical significance in the final analysis.

Gross ETE increases the stage of disease in both the AJCC-TNM staging system and ATA risk stratification. The effect of ETE on response to treatment is controversial [23, 26] but in some studies, only gross ETE has been confirmed as a predictor of recurrence [22, 27]. In our study, ETE was not a predictor of incomplete response, although gross ETE was detected in 5 patients.

In parallel to our findings, Santiago et al. reported 115 patients with low to high-risk PTC and found that age, gender, tumor size, multifocality, capsular invasion, as well as ETE are not significant predictors of response to the treatment. However, they found that high Serum Tg (OR:121.88, 95% CI:7.74 to 1918.46, *P* = 0.001) and positive antithyroglobulin after surgery (OR: 46.61, 95% CI:, *P* < 0.001) are associated with non-response to treatment [23].

In the current study, we also found that multiple RAI therapy was not associated with an ER in the majority of the patients who did not respond to the first session of treatment.

Prpic et al. reported in a study on 128 low to intermediate-risk patients with DTC who had ablation failure and needed re-ablation, a Tg level of 3.7 ng/mL before reablation predicted treatment failure but there was no association between the first, second, and cumulative dose of RAI with re-ablation outcome [30]. Hirsch et al. showed limited benefit of the second RAI therapy in DTC patients with biochemical or locoregional structural persistent disease [31]. In a retrospective review by Kaewput et al., only 16 patients (9.1%) of 176 DTC patients achieved ER at least 2 years after a cumulative dose of ≥ 600 mCi. Hence, the beneficial effects of multiple RAI therapy are controversial [32].

In this study, 16.2% of patients reached ER by the end of year three, regardless of the cumulative dose of RAI. Of a total of 36 patients who received 600 mCi or more of RAI, three patients achieved an ER by the end of the study. Although we considered various clinical, biochemical, and imaging variables and considered dynamic response to RAI-131 treatment during a three-year follow-up period, we do have some limitations. This was a single-center retrospective cohort study. Surgical procedures and ultrasound imaging were performed by different experts. The number of patients with ETE and multifocal lesions greater than 1 cm was small. A relatively low number of responders in the high-risk group limited us to explore the predictors of ER to initial RAI treatment in these patients, separately.

## Conclusion

Post-surgical stimulated serum Tg and locoregional involvement after total thyroidectomy are the main predictors of response to RAI therapy in intermediate and high-risk patients with PTC. In addition, a minority of patients achieve ER after multiple RAI therapy. Future prospective studies with a larger sample size and longer duration of follow-up are needed.

## Data Availability

The datasets used and/or analyzed during the current study available from the corresponding author on reasonable request.

## Abbreviations

ATA: American Thyroid Association
BIR: Biochemical Incomplete Response
DTC: Differentiated Thyroid Cancer
ETE: Extra Thyroidal Extension
ER: Excellent Response
IndR: Indeterminate Response
INPCR: Iranian National Population-based Cancer Registry
NR: None-Responder
NPV: Negative Predictive Value
PPV: Positive Predictive Value
PTC: Papillary Thyroid Cancer
PCLND: Prophylactic Central Lymph Node Dissection
RAI: Radioactive Iodine
R: Responder
SIR: Structural Incomplete Response
TgAb: Antithyroglobulin antibody
TT ± LND: Total Thyroidectomy ± Lymph Node Dissection
Tg: Thyroglobulin
TNM-AJCC-8: Tumor-Node-Metastasis- American Joint Committee on Cancer 8th
WBS: Whole Body Scan (RxWBS: post therapy whole-body RAI scan, DxWBS: diagnostic whole body scan)

## References

[1] Alzahrani AS, Moria Y, Mukhtar N, Aljamei H, Mazi S, Albalawi L, Aljomaiah A (2021). Course and predictive factors of incomplete response to therapy in low-and intermediate-risk thyroid cancer. J Endocr Soc.

[2] Cao CJ, Dou CY, Lian J, Luan ZS, Zhou W, Xie W, Chen L, Zhou K, Lai H (2018). Clinical outcomes and associated factors of radioiodine-131 treatment in differentiated thyroid cancer with cervical lymph node metastasis. Oncol Lett.

[3] Lubin DJ, Tsetse C, Khorasani MS, Allahyari M, McGrath M (2021). Clinical predictors of I-131 therapy failure in differentiated thyroid cancer by machine learning: a single-center experience. World J Nucl Med.

[4] Fitzmaurice C, Allen C, Barber RM, Barregard L, Bhutta ZA, Brenner H (2017). Global, Regional, and National Cancer incidence, mortality, years of Life Lost, Years lived with disability, and disability-adjusted life-years for 32 Cancer groups, 1990 to 2015: a systematic analysis for the global burden of Disease Study. JAMA Oncol.

[5] Roshandel G, Ferlay J, Ghanbari-Motlagh A, Partovipour E, Salavati F, Aryan K, Mohammadi G, Khoshaabi M, Sadjadi A, Davanlou M, Asgari F (2021). Cancer in Iran 2008 to 2025: recent incidence trends and short‐term predictions of the future burden. Int J Cancer.

[6] Haugen BR, Alexander EK, Bible KC, Doherty GM, Mandel SJ, Nikiforov YE, Pacini F, Randolph GW, Sawka AM, Schlumberger M, Schuff KG (2016). 2015 American Thyroid Association management guidelines for adult patients with thyroid nodules and differentiated thyroid cancer: The American Thyroid Association guidelines task force on thyroid nodules and differentiated thyroid cancer. Thyroid.

[7] Park HJ, Jeong GC, Kwon SY, Min JJ, Bom HS, Park KS, Cho SG, Kang SR, Kim J, Song HC, Chong A (2014). Stimulated serum thyroglobulin level at the time of first dose of radioactive iodine therapy is the most predictive factor for therapeutic failure in patients with papillary thyroid carcinoma. Nucl Med Mol Imaging.

[8] Tuttle RM, Tala H, Shah J, Leboeuf R, Ghossein R, Gonen M, Brokhin M, Omry G, Fagin JA, Shaha A (2010). Estimating risk of recurrence in differentiated thyroid cancer after total thyroidectomy and radioactive iodine remnant ablation: using response to therapy variables to modify the initial risk estimates predicted by the new American Thyroid Association staging system. Thyroid.

[9] Van Nostrand D (2017). Selected controversies of Radioiodine Imaging and Therapy in differentiated thyroid Cancer. Endocrinol Metab Clin North Am.

[10] Dong WW, Zhang DL, He L, Shao L, Wang ZH, Lv CZ, Zhang P, Huang T, Zhang H (2022). Prognostic factors for excellent response to initial therapy in patients with papillary thyroid Cancer from a prospective Multicenter Study. Front Oncol.

[11] Llamas-Olier AE, Cuéllar DI, Buitrago G (2018). Intermediate-risk papillary thyroid cancer: risk factors for early recurrence in patients with excellent response to initial therapy. Thyroid.

[12] Tamilia M, Al-Kahtani N, Rochon L, Hier MP, Payne RJ, Holcroft CA, Black MJ (2011). Serum thyroglobulin predicts thyroid remnant ablation failure with 30 mCi iodine-131 treatment in patients with papillary thyroid carcinoma. Nucl Med Commun.

[13] Matthews TJ, Chua E, Gargya A, Clark J, Gao K, Elliott M (2016). Elevated serum thyroglobulin levels at the time of ablative radioactive iodine therapy indicates a worse prognosis in thyroid cancer: an Australian retrospective cohort study. J Laryngol Otol.

[14] Yang X, Liang J, Li T, Zhao T, Lin Y (2016). Preablative stimulated thyroglobulin correlates to new therapy response system in differentiated thyroid cancer. J Clin Endocrinol Metab.

[15] Prpic M, Kust D, Kruljac I, Kirigin LS, Jukic T, Dabelic N, Bolanca A, Kusic Z (2017). Prediction of radioactive iodine remnant ablation failure in patients with differentiated thyroid cancer: a cohort study of 740 patients. Head Neck.

[16] Heemstra KA, Liu YY, Stokkel M, Kievit J, Corssmit E, Pereira AM, Romijn JA, Smit JW (2007). Serum thyroglobulin concentrations predict disease-free remission and death in differentiated thyroid carcinoma. Clin Endocrinol.

[17] Webb RC, Howard RS, Stojadinovic A, Gaitonde DY, Wallace MK, Ahmed J, Burch HB (2012). The utility of serum thyroglobulin measurement at the time of remnant ablation for predicting disease-free status in patients with differentiated thyroid cancer: a meta-analysis involving 3947 patients. J Clin Endocrinol Metab.

[18] Verburg FA, de Keizer B, Lam MG, de Klerk JM, Lips CJ, Borel-Rinkes IH, van Isselt JW (2007). Persistent disease in patients with papillary thyroid carcinoma and lymph node metastases after surgery and iodine-131 ablation. World J Surg.

[19] Creach KM, Gillanders WE, Siegel BA, Haughey BH, Moley JF, Grigsby PW (2010). Management of cervical nodal metastasis detected on I-131 scintigraphy after initial surgery of well-differentiated thyroid carcinoma. Surgery.

[20] Lepoutre-Lussey C, Maddah D, Golmard JL, Russ G, Tissier F, Trésallet C, Menegaux F, Aurengo A, Leenhardt L (2014). Post-operative neck ultrasound and risk stratification in differentiated thyroid cancer patients with initial lymph node involvement. Eur J Endocrinol.

[21] Alzahrani AS, Mukhtar N (2022). Incomplete response to therapy in intermediate-and high-risk thyroid cancer. Endocrine.

[22] Ito Y, Miyauchi A, Masuoka H, Fukushima M, Kihara M, Miya A (2018). Excellent prognosis of central lymph node recurrence-free survival for cN0M0 papillary thyroid carcinoma patients who underwent routine prophylactic central node dissection. World J Surg.

[23] Santiago AG, Isidro MJ, Parra J (2021). Predictors of response to therapy among post thyroidectomy adult Filipino patients with papillary thyroid carcinoma based on the 2015 American thyroid Association guidelines. J ASEAN Fed Endocr Soc.

[24] Pitoia F, Jerkovich F, Smulever A, Brenta G, Bueno F, Cross G (2017). Should age at diagnosis be included as an additional variable in the risk of recurrence classification system in patients with differentiated thyroid cancer. Eur Thyroid J.

[25] Moslehi M, Shahi Z, Badihian S, Jalalpour P, Sedghian M, Motamedi G, Tavakol G (2016). Differentiated thyroid cancer in Iran - initial observations, histological features, management of the disease, and tumor recurrence: a review of 1689 cases. Indian J Cancer.

[26] Póvoa AA, Teixeira E, Bella-Cueto MR, Melo M, Oliveira MJ, Sobrinho-Simões M, Maciel J, Soares P (2020). Clinicopathological features as prognostic predictors of poor outcome in papillary thyroid carcinoma. Cancers.

[27] Choi WR, Roh JL, Gong G, Cho KJ, Choi SH, Nam SY, Kim SY (2019). Multifocality of papillary thyroid carcinoma as a risk factor for disease recurrence. Oral Oncol.

[28] Mendoza ES, Lopez AA, Valdez VA, Cunanan EC, Matawaran BJ, Kho SA, Sero-Gomez MH (2016). Predictors of incomplete response to therapy among Filipino patients with papillary thyroid cancer in a tertiary hospital. J Endocrinol Invest.

[29] Ywata de Carvalho A, Kohler HF, Gomes CC, Vartanian JG, Kowalski LP (2021). Predictive factors for recurrence of papillary thyroid carcinoma: analysis of 4,085 patients. Acta Otorhinolaryngol Ital.

[30] Prpic M, Kruljac I, Kust D, Kirigin LS, Jukic T, Dabelic N, Bolanca A, Kusic Z (2016). Re-ablation I-131 activity does not predict treatment success in low- and intermediate-risk patients with differentiated thyroid carcinoma. Endocrine.

[31] Hirsch D, Gorshtein A, Robenshtok E, Masri-Iraqi H, Akirov A, Duskin Bitan H, Shimon I, Benbassat C (2018). Second radioiodine treatment: limited benefit for differentiated thyroid cancer with locoregional persistent disease. J Clin Endocrinol Metab.

[32] Kaewput C, Pusuwan P (2021). Outcomes following I-131 treatment with cumulative dose exceeding or equal to 600 mCi in differentiated thyroid carcinoma patients. World J Nucl Med.

